# Pies, Pastries, and Puddings

**Published:** 1871-01

**Authors:** 


### PIES, PASTRIES, AND PUDDINGS

Are as healthful and more nutritious than broiled steaks, roast beef, or boiled ham; and yet there will not be perhaps two readers in ten who will assent to the statement. But now that Christmas is coming, and the tables of the country will be loaded with this "forbidden fruit," it would be a great misfortune to let so many good things be neglected, but there is no danger of that, which proves that the reader practically admits the truth of the "monstrous statement" of the opening sentence, so contrary to preconceived opinion, that "pies, pastries, and puddings are as healthful, and more nutritious than broiled beefsteaks, roast turkey, or boiled ham." Nine readers in ten will regard this statement with almost contemptuous surprise. But as the year is closing in, when these things will abound in every household, let us look into the thing, with a view to eat, drink, and be merry during the grand holiday time.

Every man of science knows that there is twice as much nourishment, meaning thereby power to give warmth and strength, in a pound of sponge-cake, gingerbread, or plum-pudding, than in a pound of roast beef, for more than half the beef is water. To be specific, two thirds of the best roast beef is water, and one third nutriment at most, while puddings and "sweet cakes" of every description contain more than two thirds nutriment, and have less than one third water; hence a man will live longer on five pounds of sweet cake, than on five pounds of roast beef. Sweet cakes, pound-cakes, and the like are bread sweetened, with trifling additions, as to weight, of eggs and butter; but the flour alone out of which the cake is made, which when baked is called "bread," has all the elements of nutrition, and man can live on bread alone. But neither the eggs, nor the sugar, nor the butter, nor the milk, which added to the flour complete the ingredients of pound-cakes, are unhealthful nor innutritious; hence it is difficult to conclude that if each of half a dozen of ingredients which make up an article of food are healthful, all combined are unhealthful, espe-pecially as, if eaten singly at one meal, they would all be mixed together in the stomach, where it will be found that in the course of two or three hours after an ordinary meal, the contents of the stomach, if the owner of it is in vigorous health, are to all appearance identical in color and consistency and general appearance. The same general principles hold good as to pies and puddings. A pie is stewed fruit added to a little bread and butter; the butter being mixed with the flour before baking, making pastry, cannot by the mixture make the combination unhealthy; it will scarcely be asserted that bread and butter outside of the stomach, can become unhealthy when put inside well mixed, as they are in chewing. The same things hold good as to the prince of puddings, —

" PLUM–PUDDINGS."

The plums, the flour, the butter and the several other ingredients are healthful to eat individually; and why not collectively is difficult to comprehend.

The universal error as to the unhealthful nature of pies, puddings, and pastries, taking it for granted that they are well made and properly cooked, has arisen from the simple fact that being eaten after we have made a full meal of other things, the stomach is oppressed by them, and, if the process is repeated, becomes eventually dyspeptic; that is, has not power to work up the food, because it has been "worked to death" already. It would be quite as philosophical to say that if a man has become very tired by ploughing all day, and then by chopping wood he had "worked himself out," it was very unhealthy to chop wood.

It has frequently occurred to observant persons that having made a good meal, the unexpected appearance of a toothsome pie, or splendid pudding, or irresistible cake, instantaneously and miraculously changed their sentiments. A moment before they thought, felt, and believed that they had eaten enough; and now they believe no such thing, and forthwith eat almost as much in bulk as they had already taken, with the result very often that they are

"AS SICK AS DEATH."

Whether death is ever sick or not, is another question; but the meaning is, they feel as if they were almost sick enough to die. The legitimate conclusion is that pies, pastries, and puddings, are as healthful as roast beef or boiled tongue, if properly prepared, and eaten judiciously as to time, quantity, and quality. There are multitudes of dyspeptic persons in the common walks of life and on our farms who very rarely can afford to indulge in pies, pastries, and puddings, showing that they have become dyspeptic by eating bread and meat and vegetables at improper times, in improper quantities, or after improper preparation.

The healthy method of taking pies, pastries, puddings, and other forms of dessert is, —

First. As a luncheon, take a moderate amount and nothing else.

Second. Leave room for them at dinner-time, by eating half as much dinner as you would have done, had you known there was to be no dessert.

Third. Take your dessert at the beginning of dinner; and if any man eats too much dinner after that, he will be the greatest curiosity in existence.

